# The role of regional health policy for socioeconomic inequality in health services utilization

**DOI:** 10.1093/eurpub/ckac129.584

**Published:** 2022-10-25

**Authors:** I Iashchenko, W Schüttig, P Bammert, J Spallek, P Rattay, S Schneider, I Moor, CR Pischke, L Sundmacher

**Affiliations:** 1 Technical University of Munich, Department of Sport and Health Sciences, Munich, Germany; 2 Brandenburg University of Technology, Department of Public Health, Senftenberg, Germany; 3 Robert Koch-Institute, Department of Epidemiology and Health Monitoring, Berlin, Germany; 4 Heidelberg University, Center for Preventive Medicine and Digital Health, Mannheim, Germany; 5 Martin Luther University Halle-Wittenberg, Institute of Medical Sociology, Halle, Germany; 6 Heinrich-Heine University Düsseldorf, Child Health Services Unit, Düsseldorf, Germany

## Abstract

**Background:**

“J1” is a preventative routine examination in Germany recommended for adolescents at the age of 12-14 years. In contrast to the well-established U1-U9 examinations for younger children, with participation rates above 90%, the attendance of the J1 examination is approximately only 40%. The most frequent reason for not attending J1 is the unawareness of this examination. “Ticket to J1” is an intervention including an information leaflet introduced in Bavaria in 2017 to inform adolescents about J1. The aims of the present analysis are to investigate (1) if the regional policy was effective in increasing the attendance in J1, (2) if the effects vary by family socioeconomic status (SES), and (3) which meso-level characteristics of the healthcare system correlate with attendance rates in J1.

**Methods:**

We used anonymised data of a large statutory health insurance in Germany for the timeframe of 2016-2018. To investigate the effect of the policy, a difference-in-differences design at the individual level was used. Assuming a parallel trend at the level of federal states, the likelihood of attendance in J1 of 13- and 14-year-olds was compared between Bavaria and other federal German states before and after policy introduction. All analyses were additionally stratified by SES.

**Results:**

The introduction of “Ticket to J1” increased participation in J1 by 1% after controlling for all confounders. Furthermore, the effect was stronger for children from families with lower SES (an increase of 5%). Density of pediatricians was positively significantly correlated with participation in J1.

**Discussion:**

Regional health policy intervention had a significant positive impact on attendance of J1 and appears to have the potential to reduce socioeconomic inequalities in healthcare utilization. Informing adolescents about J1 seems to increase the attendance, in particular for children from families with lower SES.

